# Automated sampling and analysis in research product synthesis

**DOI:** 10.1155/S1463924692000312

**Published:** 1992

**Authors:** L. Timmermans, A. Van den Bergh, A. Verhecken, C. Van de Sande

**Affiliations:** PRO-CBP Agfa-Gevaert N. V. Septestraat 27 Mortsel 2640 Belgium

## Abstract

The information obtained about relevant reaction parameters can be greatly increased by monitoring concentration changes during a reaction. To achieve this goal, a fully automated system was designed which handles both sampling and analysis. The sampling system takes samples at predefined intervals, and also performs a number of tasks such as dilution, neutralization, filtration and analysis.

The examples show the universal applicability of the device regarding to solvents, reaction media and reaction type. It is also demonstrated that the information, included in the concentration profiles, greatly increases our knowledge about the reaction. This increase in information, in conjunction with other data,for example calorimetry, could be used for reaction simulation software.


Journal of Automatic Chemistry, Vol. 14, No. 5 (September–October 1992), pp. 169–172

### Automated sampling and analysis in research product synthesis

**L. Timmermans, A. Van den Bergh, A. Verhecken and C. Van de Sande**
*PRO-CBP, Agfa-Gevaert N.V., Septestraat 27, 2640 Mortsel, Belgium*

*The information obtained about relevant reaction parameters can be greatly increased by monitoring concentration changes during a reaction. To achieve this goal, a fully automated system was designed which handles both sampling and analysis. The sampling system takes samples at predefined intervals, and also performs a number of tasks such as dilution, neutralization, filtration and analysis.*

*The examples show the universal applicability of the device regarding to solvents, reaction media and reaction type. It is also demonstrated that the information, included in the concentration profiles, greatly increases our knowledge about the reaction. This increase in information, in conjunction with other data, for example calorimetry, could be used for reaction simulation software.*

### Introduction

Sampling devices have been developed for many applications, mostly dedicated ones [1–4]. The idea of developing an automated sampling and analysis device as an aid for the optimization and scaling-up of research product synthesis resulted from the need to monitor concentration changes during a reaction.

When discussing the requirements for the automated sampling device, it soon became clear that a lot of requirements had to be satisfied for the device to be universally applicable:

(1) It should be possible to process all types of solvents and reaction media (solutions, emulsions, suspensions).
(2) The whole sampling and analysis procedure should be fully automated – after the operator has started an experiment, all manipulations should be automatically performed.
(3) The execution time of the sampling procedure should be kept to a minimum to ensure that short reactions are possible.
(4) Because many reactions are carried out in the presence of salts which do not dissolve in the dilution solvent, there must be some provision for on-line filtration of the diluted sample.
(5) Sampling and analysis procedures should be able to run simultaneously. Long analysis times should not interfere with sampling times so that sampling time is not governed by the analysis time.

A pump is used to carry the sample to the mixing chamber, so a system which signalled the need to stop the pump had to be established. A conductivity-based sensor with platinum probes molten in the inlet was developed for this purpose. This allows a large number of solvents to be detected, including methylene chloride.

### Materials

The instrumentation used for the automated sampling device consists of an automated reactor (Contalab), an FMI pump (model QC 216, Fluid Metering Inc.), a sampling device (see figure 1). a magnetic stirrer (Telesystem Micro, VarioMag), a timer (Syrelec 1000 PA), three-way solenoid valves (General Valve Corporation A280), HPLC apparatus (autoinjector 231, dilutor 401, pump 305, pump 306, UV detector 116, mixing chamber and a manometric module, all from Gilson), HPLC software (GME 715, Gilson) and a PC (PS/2 model 50 IBM).

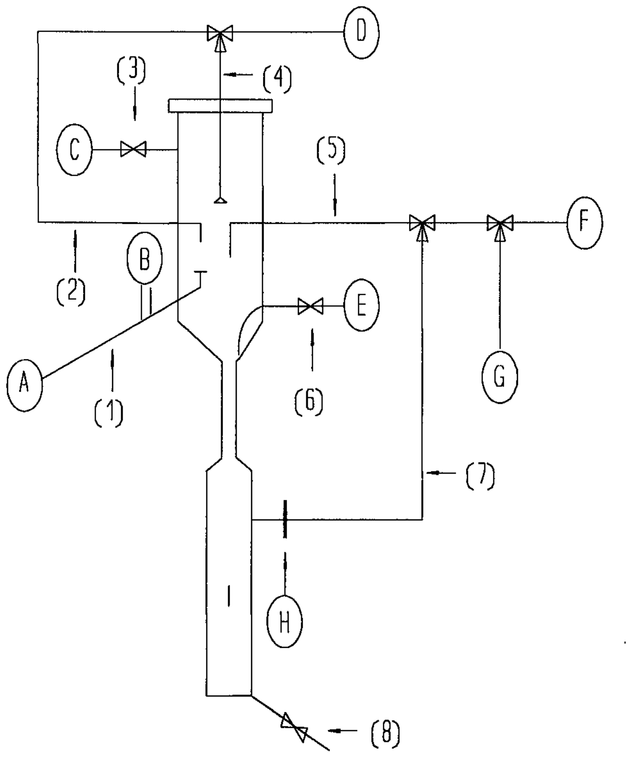

*Figure 1. Schematic set-up for the automated sampling device. A = to FMI pump; B = conductivity sensor; C = nitrogen pressure; D = to dilutor 0; E = vacuum suction; F = to dilutor 1; G = to vials; H = in-line filter; I = mixing chamber; 1 = inlet sample; 2 = rinse inlet sample with reaction solvent; 3 = line for nitrogen pressure; 4 = rinse mixing chamber with dilution solvent; 6 = line for suction of excess sample; 7 = in-line filtration of the diluted sample; 8 = drain for excess diluted sample.*

0142–0453/92 $3.00 © 1992 Taylor & Francis Ltd.

The HPLC columns used were: Merck, LichroCart, RP-18 5 μm 100 Å, L = 125 mm d = 4 mm; and Machery & Nagel, Nucleosil, kieselgel 5 μm 60 Å, L = 10 mm d = 4.6 mm.

### Working procedure

A number of tasks must be fulfilled before each experiment. For all the relevant components, calibration curves must be created in order to obtain quantitative information. Next, a suitable solvent in which the reagents and products dissolve readily should be found. At the start of each experiment, the tubings must be filled according to their destination either with reaction solvent or with dilution solvent as shown in figure 1. Finally, the dilution factor and mixing time must be set.

The mixing chamber is then filled with exactly 28 ml of dilution solvent. The dilution solvent used obviously needs to be able to dissolve possible precipitates. The sample is pumped into the mixing chamber through the inlet tubing. About 0·9 ml of sample is added. Mixing of sample and dilution solvent is initially prevented by means of the narrow glass tube in the middle of the apparatus.

The inlet tubing (1 in figure 1) is rinsed with reaction solvent through line (2) after excess sample is sucked away through line (6) by means of vacuum. Then the dilution solvent and sample are thoroughly mixed with a magnetic stirrer for as long as indicated by the preset mixing time. This is especially important when dealing with suspensions. The diluted sample is led via the in-line filter (7) to a vial on the rack of the autoinjector where it is further diluted according to the preset dilution factor. Samples can be stored as long as the dilution is sufficient to quench the progression of the reaction in stored samples. This allows an analyis to be repeated as many times as needed and a sample to be taken during the analysis of an earlier sample (sampling time < analysis time). The excess of sample left in the mixing chamber is drained through line (8) by means of nitrogen pressure (3). The mixing chamber is rinsed twice with reaction solvent (4) and blown dry with nitrogen gas (3). This ensures a clean and dry apparatus for the next sample. Finally, the sample is injected into the HPLC and analysis starts.

### Experimental

### *Sampling reproducibility and accuracy*

In order to establish the sampling reproducibility and accuracy, a standard solution of 0.1 M nitroindazole in methoxypropanol was prepared. The reactor was filled with 1 l of the standard solution and maintained at room temperature. Then 10 samples were taken and subsequently analysed. As the samples are diluted 28·9 times in the mixing chamber, and are not further diluted in the vials, this corresponds to a concentration of 0·50 mg/ml of nitroindazole in the mixing chamber. The analysis results are summarized in table 1. The experimental concentration is 0·51 mg/ml ± 0·02 mg/ml. These values are perfectly acceptable to most applications. For a reaction it is also possible to calculate the concentration by means of multiplication of the fraction of the component with the initial concentration of the relevant reagent. In this way, errors are cancelled in the calculation of the fractions.

*Table 1. Results of the reproducibility and accuracy tests for the sampling procedure. The response factor for nitroindazole is 2·17 × $10^6$.*

| Sample | Counts | Concentration (mg/ml) mixing chamber |
|---|---|---|
| 1 | 1107250 | 0·51 |
| 2 | 1037251 | 0·48 |
| 3 | 1084754 | 0·50 |
| 4 | 1018951 | 0·47 |
| 5 | 1167017 | 0·54 |
| 6 | 1154191 | 0·53 |
| 7 | 1118291 | 0·51 |
| 8 | 1138411 | 0·52 |
| 9 | 1088837 | 0·50 |
| 10 | 1128385 | 0·52 |

### *Acid-catalysed hydrolysis of an ester (see figure 2)*

Hydrolysis was carried out in refluxing methoxypropanol in the presence of sulphuric acid, diluted with demineralized water. The catalyst was added after the solution reached reflux temperature. From then on, for a total of about 7 h, samples were taken every 15 min. Because each HPLC analysis took about 10 min, each sample was analysed before the next sample was taken. The end-point for the reaction could thus be readily detected. In order to demonstrate the usefulness of the apparatus in optimization studies, this experiment was repeated with (1) double acid catalyst concentration and with (2) double acid catalyst and halved ester concentration. Figure 3 shows the concentration profiles for the first experiment.

### *Oxidation of an aromatic sulphide (see figure 4)*

The oxidation was carried out in acetic acid/water in the absence of a catalyst. The 30% hydrogen peroxide solution was added in two steps with an interval of 30 min between the two additions. Samples were taken from the emulsion every 10 min during the first step (sulphoxide) and every 15 min during the second step (sulphone). Because the HPLC analysis took about 45 min, the end of the reaction could not be readily detected; hence samples were taken for about 7 h. This working procedure is quite manageable because it is possible to store the samples.

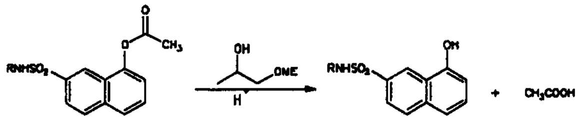

*Figure 2. Acid-catalysed hydrolysis of an ester.*

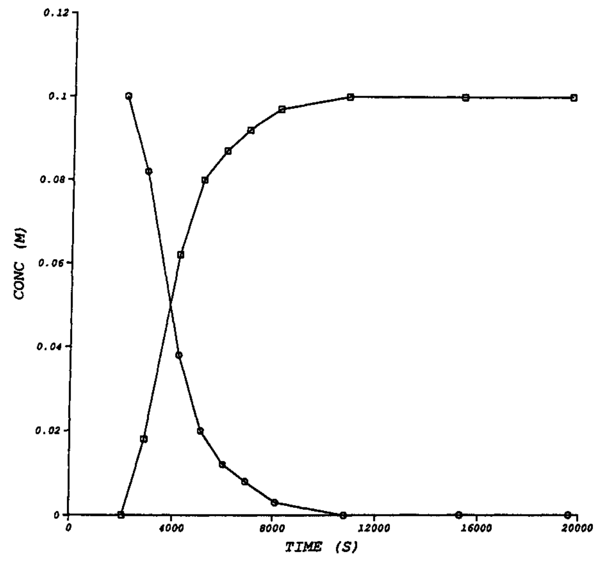

*Figure 3. Concentration profiles for the acid catalysed hydrolysis of an ester at reflux. ● = starting material; ■ = product.*

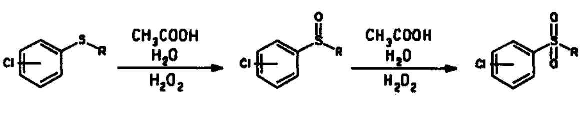

*Figure 4. Oxidation of an aromatic sulphide at 90 °C.*

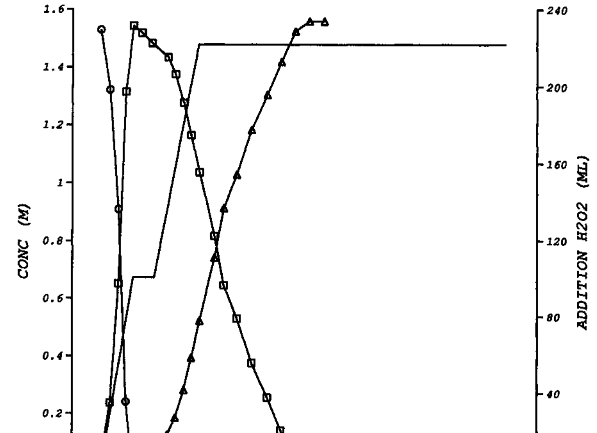

*Figure 5. Concentration profiles for the oxidation of an aromatic sulphide at 90 °C. ● = sulphide; ■ = sulphoxide; ▲ = sulphone.*

This experiment was carried out at 60 °C and at 90 °C. Figure 5 shows the concentration profiles for the second experiment.

When these experiments are compared, it is clear that the first oxidation step follows the addition of the hydrogen peroxide, whereas the second oxidation step does not. There is also some sulphone formed before the second addition of the hydrogen peroxide is started.

*Condensation of an aldehyde with a pyrazolone (see figure 6)*

The condensation was carried out in refluxing methanol in the presence of sodium acetate as the base. Because of the great polarity of pyrazolone and its condensation product, due to the presence of sulphonic and carboxylic groups, analysis was performed on a normal phase column with 85% methanol, 10% 0·5 M NaCl and 5% 0·03 M $Na_2HPO_4$ pH 8 as the eluent. This ensured an analysis time of 5 min. Samples were taken from the solution every 10 min at the start of the experiment and every 30 min after a few hours of reaction. This continues for a total of about 6 h. The end-point of the reaction is readily detected because of the short analysis time. As a variant, the order of addition of the reagents in the reactor was changed. This raised problems with the dilution because it was impossible to find a solvent that dissolved the compounds in an acceptable time. Figure 7 shows the concentration profiles for this reaction.

*Condensation of an anhydride with a substituted indazole (see figure 8)*

The condensation was carried out in dry acetone with triethylamine acting as the base. It was known from earlier experiments that two isomers were formed as reaction products. For one of these isomers it was impossible to create a calibration curve because the isomer was not available as a sufficiently pure chemical. Samples were taken from the dense suspension every 9 min at the start of the experiment and every 30 min after a few hours of reaction. This continued for a total of about 7 h.

This experiment was repeated four times, each time at a different reaction temperature (reflux, 40 °C, 20 °C, 10 °C). As can clearly be seen in figure 9, which shows the concentration profiles for the experiment at 20 °C, a 7 h reaction time is much too long as the reaction is terminated after 90 min. As the temperature decreases, the fraction of the second isomer increases strongly. It would be interesting to carry out the reaction with non-stoichiometric amounts as an optimization experiment. Note that the second isomer is formed at the expense of the first isomer.

### Discussion

The examples given in the experimental section show that the automated sampling device is not dedicated to particular applications, but rather, is widely applicable. All types of reaction media can be processed: solutions, emulsions and suspensions. It is also possible to store the samples for later manipulation.

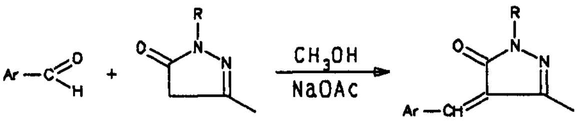

*Figure 6. Condensation of an aldehyde with a pyrazolone.*

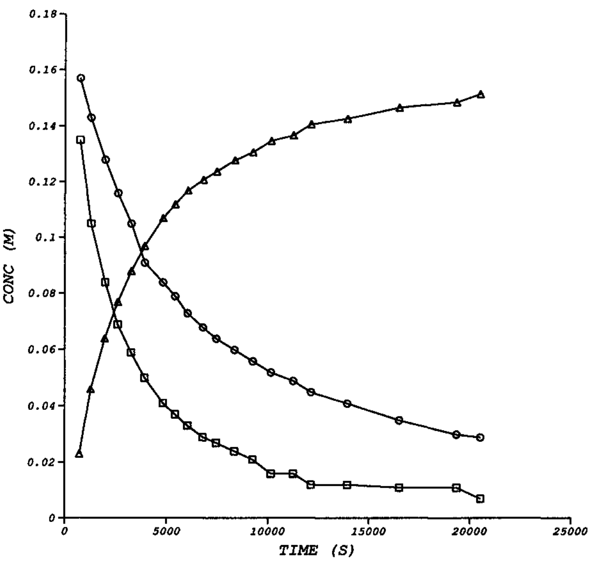

*Figure 7. Concentration profiles for the condensation of an aldehyde with a pyrazolone at reflux. ● = aldehyde; ■ = pyrazolone; ▲ = product.*

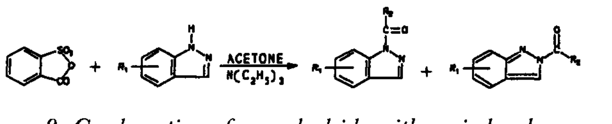

*Figure 8. Condensation of an anhydride with an indazole.*

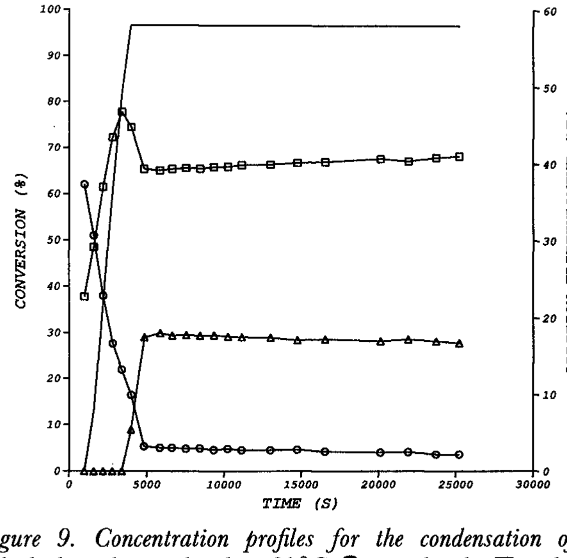

*Figure 9. Concentration profiles for the condensation of an anhydride with an indazole at 20 °C. ● = indazole; ■ and ▲ = products.*

With the fully automated feature, there is no need for an extra person to manage the device – the reactor's operator can simultaneously supervise the sampling apparatus.

The main goal of the automated sampling device, i.e. increasing the information-gain per reactions, has clearly been achieved. With other methods only overall reaction information, such as calorimetric results, is given, whereas here the concentration profiles represent specific component bounded information.

The information included in these concentration profiles includes:

(1) The influence of an increase in concentration of the catalyst.

(2) The rate determining step in a consecutive reaction.

(3) Whether oxidation follows the addition of the oxidant?

(4) Whether it is necessary to reflux for so long?

(5) The ideal temperature to carry out the reaction in order to hold the concentration of a byproduct at a minimum?

This information should make it possible to carry out the reactions in a more reproducible manner. Ultimately, it should be possible to combine concentration profile data and thermochemical data in reaction simulation software so as to deduce optimum reaction conditions by modelling, as opposed to performing series of traditional experiments.

### Acknowledgements

The authors wish to thank L. Jennis, the glassblower who meticulously and with great patience, made the sampling device. In addition, thanks are due to colleagues who provided the reactions to study.

### References

1. Turnell, D. C. and Cooper, J. D. H., *Journal of Automatic Chemistry*, **4** (1985), 177–180.
2. Favre, E., Pugeaud, P., Raboud, J. P. and Peringer, P., *Journal of Automatic Chemistry*, **6** (1989), 280–283.
3. Kramer, G. W. and Fuchs, P. L., *Chemtech*, **19** (1989), 682–688.
4. Tena, M. T., Luque de Castro, M. D. and Valcarcel, M., *Journal of Automatic Chemsitry*, **3** (1991), 111–113.

